# A Practical Treatise on Inflammation, &c., of the Uterus and Its Appendages

**Published:** 1850-11

**Authors:** 


### ARTICLE VII.

*A practical Treatise on Inflammation of the Uterus and its appendages, and on Ulcerations and indurations of the neck of the Uterus.*—By JAMES HENRY BENNETT, M.D., Member of the Royal College of Physicians, etc. Second American, from the second London edition. pp. 355; 8vo. Philadelphia: Lea & Blanchard, 1850. (From the Publishers, through J. Keen, Jr. & Bro.)

This work, which has attracted so much attention both in this country and Europe, on account of the, previously unsuspected, frequency of ulceration of the cervix Uteri which it asserts, is well worthy the attention of the profession. The frequent practice of vaginal examinations with the speculum, called for by the doctrines taught in this work, has met the reprehension of Dr. R. Lee and other eminent obstetricians; but the whole argument seems to result in a conclusion that the use of the speculum ought not to be abused. It is clear that too frequent use of the speculum, would be by no means as serious an error, as an omission of its use where it was actually necessary to develope the true nature of the case.

